# Associations between circadian alignment and cognitive functioning in a nationally representative sample of older adults

**DOI:** 10.1038/s41598-024-64309-9

**Published:** 2024-06-12

**Authors:** Sophie Leahy, Qian Xiao, Chris Ho Ching Yeung, Mariana G. Figueiro

**Affiliations:** 1 Department of Population Health Science and Policy, Light and Health Research Center, Icahn School of Medicine at Mount Sinai, New York, NY USA; 2 Department of Epidemiology, Human Genetics, and Environmental Sciences, School of Public Health, The University of Texas Health Science Center at Houston, Houston, TX USA; 3 School of Public Health, Center of Spatial-Temporal Modeling for Applications in Population Sciences, The University of Texas Health Science Center at Houston, Houston, TX USA

**Keywords:** Circadian rhythms and sleep, Cognitive neuroscience

## Abstract

Proper alignment of activity-rest and light–dark patterns allows for healthy bodily functions to occur at optimal times of the day. Disruptions to this alignment may cause poor sleep as well as physical, mental, and cognitive problems. The purpose of this cross-sectional study was to determine if poorer circadian alignment was associated with decreased cognitive functioning among older (> 60 years) participants in the National Health and Nutrition Examination Survey. We utilized actigraphy-based rest-activity and dark-light measurements to calculate phasor magnitude (strength of circadian alignment coupling) and phasor angle (phase difference between activity-rest and light–dark cycles). Multiple linear regression models were used to determine associations of phasor magnitude and angle with performance in various cognitive tests, including Digit Symbol Substitution Test score (DSSS), CERAD Savings Percentage (CSP), and Animal Fluency Test (AFT) score. The results showed that a lower phasor magnitude (which indicates decreased strength of alignment coupling between rest-activity and dark–light cycles) was significantly associated with decreased DSSS (indicating slower processing speed and poorer working memory) when controlling for many important sociodemographic factors. However, this association became non-significant when accounting for sleep duration and total physical activity. Phasor angle did not have a significant association with any of the cognitive scores. Overall, we provided evidence indicating that circadian alignment may be a predictor of cognitive performance. Future studies should investigate whether improving circadian alignment may improve cognitive function and prevent cognitive decline.

## Introduction

Cognitive impairment and dementia have become increasingly prevalent in older adults in the United States (US). From 1996 to 2014, cognitive impairment increased from 19 to 21% in women over 50, and from 18 to 21% in men over 50^1^. Furthermore, it is estimated that the number of people in the US suffering from mild cognitive impairment (MCI) and Alzheimer’s disease and related dementias (ADRD) will increase from 12.2 million in 2020 to 21.6 million in 2060^2^. Identifying modifiable risk factors of cognitive diseases is an important step in establishing public health initiatives and clinical interventions to prevent cognitive decline and promote healthy aging.

Many researchers^3,4^ have recognized that sleep and circadian rhythm play an important role in both physiological and cognitive functions. Xiao et al.^5,6^ established that less stable and more variable rest-activity rhythms, as well as reduced rest-activity amplitude and later activity timing, were associated with incident cognitive impairment and dementia in older men and women. In addition, jet lag and nightshift work, which often lead to severe sleep and circadian disruption, have been linked to cognitive impairments and reduced memory function^7–13^. These findings indicate that sleep and circadian disruption may play a role in cognitive decline.

Light–dark patterns reaching the retina send day-night information to the master circadian clock in the suprachiasmatic nucleus (SCN)^14–16^. This information is processed by the SCN and promotes optimal alignment between the timing of the SCN and the external environment, so physiological functions, including sleep, occur at optimal times of day. This process is known as entrainment^17^. Lack of entrainment, or circadian disruption, has been associated with various maladies such as reduced insulin sensitivity, cardiovascular dysfunction, and sleep and mood disorders^18–26^. Similarly, night shift workers commonly suffer from circadian disruption, which has been found to reduce executive function, sustained attention, visual motor performance, and processing times^10,13^. Khan et al. performed a cross-sectional analysis using the baseline Canadian Longitudinal Study on Aging database, including 47,811 middle-aged and older adults (45–85 years)^11^. They found that exposure to night shift work was associated with overall cognitive impairment, and that longtime night shift work was associated with memory function impairment. Compared to day shift workers, those working rotating shifts had greater impairment in executive function measures. Studies of airline cabin crew members have shown that jet lag causes deficits in spatial learning and working memory^7,8^. Further, groups that compared day shift versus night shift work found that night shift work was associated with more severe cognitive impairment^11,12^.

In the current scope of available research, one study has correlated alignment measurements with executive cognitive functioning in a sample of largely white and college-educated older males^27^. The present study aims to broaden the understanding of the impact of circadian disruption by addressing alignment and cognition in a more diverse sample of older men and women, including a larger percentage of participants from non-white races and ethnicities as well as lower socioeconomic backgrounds.

Using a large and nationally representative sample of American adults through the National Health and Nutrition Examination Survey (NHANES) database with multi-day 24-h measurements of activity and light, we applied phasor analysis to quantify two major aspects of the alignment between rest-activity and dark–light cycles: (1) the strength of coupling (i.e., phasor magnitude) and (2) the temporal relationship (i.e., phasor angle) between these two variables. More specifically, smaller phasor magnitude may imply weaker entrainment because the correlation of the light and activity patterns is low. Meanwhile, larger phasor angles indicate greater temporal distance between these patterns. We examined phasor magnitude and angle in relation to various domains of cognitive functioning, including processing speed, working memory, sustained attention, and verbal fluency. The purpose of the current analysis was to test the hypotheses that cognitive impairments are associated with (1) decreased phasor magnitudes and (2) larger phasor angles. Because the NHANES database contains a large and diverse sample, we also examined whether the relationship between phasor magnitude and angle and cognitive function differs according to demographic and lifestyle factors.

## Results

Table 1 presents descriptive statistics by quintiles of phasor magnitude. There was a general trend of decreased percentages of Black participants and participants with an income of less than $20,000 as phasor magnitude increased. Meanwhile, there were higher percentages of married participants, current drinkers, and people who slept fewer than 7 h per night as phasor magnitude increased. Additionally, total physical activity increased with higher phasor magnitude. Phasor angle decreased with higher phasor magnitude, starting at Q2.

**Table 1:** Selected study characteristics by quintiles of phasor magnitude in adults in NHANES 2011–2014.

Characteristic	Phasor magnitude quintile^a^					*P* value^b^
	Q1	Q2	Q3	Q4	Q5	
Phasor magnitude (mean ± SE)	0.12 ± 0.002	0.21 ± 0.001	0.27 ± 0.001	0.34 ± 0.001	0.45 ± 0.003	–
Phasor angle (hour, mean ± SE)	0.70 ± 0.10	0.70 ± 0.07	0.57 ± 0.07	0.46 ± 0.05	0.42 ± 0.05	0.007
Age (years, mean ± SE)	71.2 ± 0.5	71.2 ± 0.4	69.5 ± 0.3	69.0 ± 0.4	68.0 ± 0.4	0.001
Female (%)	54.1	56.3	54.9	56.0	55.0	0.949
Race/ethnicity (%)						< 0.001
Non-Hispanic white	73.7	72.5	75.5	78.5	86.0	–
Non-Hispanic Black	14.3	11.3	10.9	7.8	3.8	–
Hispanic	5.9	7.8	8.2	7.1	7.5	–
Other	6.2	8.4	5.4	5.6	3.1	–
Less than high school (%)	22.2	18.6	19.3	19.7	15.2	0.299
Household income < $20,000 (%)	25.9	22.8	18.8	16.9	10.4	< 0.001
Married (%)	49.7	53.3	61.3	62.0	71.8	< 0.001
Current smoker (%)	18.3	9.0	9.6	10.6	9.4	0.06
Current alcohol drinker (%)	50.6	54.5	62.6	66.6	71.5	< 0.001
Sleep duration, hours (%)						< 0.001
< 7 h	34.9	34.3	45.4	48.4	49.6	–
7–9 h	35.7	43.8	44.9	42.3	45.9	–
> 9 h	29.4	21.9	9.7	9.2	5.5	–
Total physical activity^c^ (mean ± SE)	7040 ± 220	8161 ± 125	8880 ± 197	9925 ± 133	11,029 ± 137	< 0.001

*SE* standard error, *NHANES* National Health and Nutrition Examination Survey.

^a^Means, standard errors, and percentages are weighted using sample weights.

^b^*P* values reflect results of ANOVA tests for continuous variables and X^2^ tests for categorical variables.

^c^Measured as the daily sum of per-minute Monitor-Independent Movement Summary values.

Table 2 presents descriptive statistics by phasor angle quintile. Higher phasor angle was associated with younger age. There was a trend of increased percentage of participants that did not finish high school, those that had an income of less than $20,000, and those who currently smoked, with increased phasor angle, starting at Q2. There was also a trend of decreased phasor magnitude and decreased percentage of white and married participants with increased phasor angle quintile, starting at Q2.

**Table 2:** Selected study characteristics by quintiles of phasor angle in adults in NHANES 2011–2014.

Characteristic	Phasor angle quintile^a^					*P* value^b^
	Q1	Q2	Q3	Q4	Q5	
Phasor magnitude (mean ± SE)	0.28 ± 0.01	0.32 ± 0.01	0.32 ± 0.01	0.30 ± 0.01	0.23 ± 0.01	< 0.001
Phasor angle (hour, mean ± SE)	− 0.78 ± 0.04	0.14 ± 0.01	0.61 ± 0.01	1.13 ± 0.01	2.22 ± 0.04	–
Age (years, mean ± SE)	70.8 ± 0.4	70.0 ± 0.3	69.1 ± 0.3	69.0 ± 0.4	69.1 ± 0.3	0.005
Female (%)	49.0	55.1	58.7	58.2	54.6	0.035
Race/ethnicity (%)						< 0.001
Non-Hispanic white	81.9	83.7	81.0	72.5	66.7	–
Non-Hispanic Black	8.2	7.2	8.3	10.3	12.5	–
Hispanic	4.4	5.5	7.0	10.6	11.6	–
Other	5.5	3.7	3.7	6.6	9.2	–
Less than high school (%)	16.0	15.6	16.9	22.8	24.6	0.033
Household income < $20,000 (%)	17.6	14.6	16.2	19.1	25.4	0.04
Married (%)	62.0	66.8	61.7	57.8	52.7	0.004
Current smoker (%)	12.9	7.2	8.5	11.3	17.4	0.013
Current alcohol drinker (%)	62.3	63.7	64.8	61.2	58.5	0.606
Sleep duration, hours (%)						0.474
< 7 h	41.3	41.0	42.8	47.1	46.7	–
7–9 h	43.7	4.6	43.1	38.3	42.6	–
> 9 h	15.0	14.4	14.1	14.7	10.7	–
Total physical activity^c^ (mean ± SE)	8914 ± 201	9560 ± 122	9225 ± 132	9476 ± 139	8857 ± 198	0.0103

*SE* standard error, *NHANES* National Health and Nutrition Examination Survey.

^a^Means, standard errors, and percentages are weighted using sample weights.

^b^*P* values reflect results of ANOVA tests for continuous variables and X^2^ tests for categorical variables.

^c^Measured as the daily sum of per-minute Monitor-Independent Movement Summary values.

Table 3 presents associations between phasor magnitude and cognitive measures. These cognitive measures include the Digit Symbol Substitution Test score (DSSS), which measures processing speed and attention^28^; the Consortium to Establish a Registry for Alzheimer’s Disease (CERAD) test scores and CERAD Savings Percentage (CSP), which test for memory and information retention^29–31^; and the Animal Fluency Test (AFT), which assesses verbal fluency^32^. Results from the minimal model (Model 1) showed that a lower phasor magnitude was associated with worse DSSS and AFT score (*P* trends < 0.001 and < 0.003, respectively). After controlling for additional confounders, results from the main model (Model 2) showed that lower phasor magnitude remained associated with worse DSSS only (b_Q1 v. Q5_ (95% CI) (− 3.89 (− 6.27, − 1.52)), (*P* trend = 0.014). Finally, after controlling for sleep duration and total physical activity (Model 3), no associations between phasor magnitude and cognitive scores remained statistically significant. In the analyses focusing on immediate and delayed CERAD recall scores (Supplementary Table 1), the minimal model (Model 1) showed that lower phasor magnitude was associated with worse performance in both immediate and delayed recall (*P* trends = 0.018 and 0.031, respectively), whereas the associations were attenuated after controlling for potential confounders (Model 2) and sleep and physical activities (Model 3).

**Table 3:** Associations of phasor magnitude and with cognitive test scores in NHANES 2011–2014.

Phasor magnitude quintile	Cognitive test scores^a^			
	Mean ± SE	Beta estimates (95% CI)		
		Model 1	Model 2 (main)	Model 3
DSSS				
Q1	46.5 ± 1.1	− 7.24 (− 9.59, − 4.90)	− 3.89 (− 6.27, − 1.52)	− 1.97 (− 4.02, 0.08)
Q2	49.8 ± 1.0	− 3.90 (− 7.13, − 0.66)	− 1.65 (− 4.18, 0.88)	− 0.32 (− 2.70, 2.05)
Q3	52.1 ± 1.0	− 2.86 (− 5.52, − 0.19)	− 0.65 (− 2.95, 1.64)	0.30 (− 1.86, 2.47)
Q4	51.7 ± 1.2	− 3.64 (− 6.38, − 0.90)	− 1.87 (− 4.06, 0.33)	− 1.35 (− 3.43, 0.73)
Q5	56.4 ± 1.1	Ref^b^	Ref	Ref
*P* trend	–	< 0.001	0.014	0.307
CSP				
Q1	74.7 ± 2.1	− 3.83 (− 8.55, 0.89)	− 2.94 (− 7.42, 1.55)	− 1.22 (− 6.43, 3.99)
Q2	76.7 ± 1.6	− 1.60 (− 5.53, 2.32)	− 1.43 (− 5.64, 2.79)	− 0.27 (− 4.95, 4.41)
Q3	77.6 ± 1.3	− 2.08 (− 6.00, 1.84)	− 1.60 (− 5.58, 2.38)	− 0.77 (− 4.86, 3.31)
Q4	79.7 ± 1.3	− 0.33 (− 3.11, 2.45)	− 0.18 (− 3.20, 2.85)	0.28 (− 2.71, 3.26)
Q5	80.9 ± 1.0	Ref	Ref	Ref
*P* trend	–	0.131	0.212	0.657
AFT score				
Q1	16.8 ± 0.4	− 1.31 (− 2.25, − 0.38)	− 0.53 (− 1.26, 0.20)	0.06 (− 0.74, 0.86)
Q2	17.3 ± 0.3	− 0.76 (− 1.47, − 0.05)	− 0.25 (− 0.93, 0.44)	0.19 (− 0.57, 0.95)
Q3	17.9 ± 0.3	− 0.62 (− 1.41, 0.18)	− 0.20 (− 0.90, 0.50)	0.10 (− 0.57, 0.77)
Q4	18.3 ± 0.4	− 0.27 (− 1.02, 0.48)	0.06 (− 0.60, 0.72)	0.22 (− 0.44, 0.87)
Q5	18.8 ± 0.3	Ref	Ref	Ref
*P* trend	–	0.003	0.116	0.870

Model 1: adjusted for age and gender.

Model 2: adjusted for variables in Model 1 and race/ethnicity, education, household income, marital status, smoking, and alcohol consumption.

Model 3: adjusted for variables in Model 2 and sleep duration and total physical activity.

*CI* confidence interval, *NHANES* National Health and Nutrition Examination Survey, *CERAD* Consortium to Establish a Registry for Alzheimer’s Disease, *DSSS* Digit Symbol Substitution Test score, *CSP* CERAD Savings Percentage, *AFT* Animal Fluency Test.

^a^Means, standard errors, and beta estimates are weighted using sample weights.

^b^Reference group presumed as having the best light–dark and activity-rest coupling.

Table 4 presents the association between phasor angle and cognitive scores. A more delayed phasor angle was associated with worse performance on the DSST in the analysis based on minimal model (*P* trend ≦ 0.001), but the association was attenuated and became not statistically significant after controlling for other confounders. No association between phasor angle and CSP or AFT score were observed. Phasor angle was associated with immediate and delayed CERAD recall in the minimal model (Model 1); however, these associations became non-significant when other variables were controlled for in Models 2 and 3 (Supplementary Table 2).

**Table 4:** Associations of phasor angle and with cognitive test scores in NHANES 2011–2014.

Phasor angle quintile	Cognitive test scores^a^			
	Mean ± SE	Beta estimates (95% CI)		
		Model 1	Model 2 (main)	Model 3
DSSS				
Q1	52.5 ± 1.1	Ref^b^	Ref	Ref
Q2	52.7 ± 0.8	− 1.00 (− 3.02, 1.03)	− 1.50 (− 3.28, 0.29)	− 1.67 (− 3.39, 0.05)
Q3	52.7 ± 0.7	− 2.04 (− 4.35, 0.28)	− 2.09 (− 3.87, − 0.32)	− 2.00 (− 3.65, − 0.36)
Q4	50.6 ± 1.2	− 4.15 (− 6.83, − 1.48)	− 2.08 (− 4.06, − 0.10)	− 2.10 (− 4.09, − 0.11)
Q5	50.4 ± 1.1	− 4.57 (− 6.68, − 2.46)	− 1.40 (− 3.07, 0.28)	− 1.35 (− 3.06, 0.36)
*P* trend	–	< .0001	0.078	0.109
CSP				
Q1	78.7 ± 1.5	Ref	Ref	Ref
Q2	77.4 ± 1.1	− 2.37 (− 6.54, 1.79)	− 2.83 (− 6.83, 1.17)	− 3.03 (− 7.07, 1.00)
Q3	77.4 ± 1.7	− 3.19 (− 8.04, 1.67)	− 3.46 (− 8.34, 1.41)	− 3.39 (− 8.19, 1.41)
Q4	79.5 ± 1.4	− 1.20 (− 5.09, 2.70)	− 0.83 (− 4.56, 2.91)	− 0.87 (− 4.62, 2.88)
Q5	78.9 ± 1.0	− 1.85 (− 5.80, 2.10)	− 1.17 (− 5.13, 2.79)	− 1.16 (− 5.01, 2.68)
*P* trend	–	0.486	0.827	0.834
AFT score				
Q1	17.8 ± 0.3	Ref	Ref	Ref
Q2	18.2 ± 0.3	0.16 (− 0.68, 1.00)	0.07 (− 0.74, 0.88)	0.04 (− 0.76, 0.85)
Q3	18.0 ± 0.3	− 0.18 (− 0.99, 0.63)	− 0.18 (− 0.94, 0.58)	− 0.14 (− 0.88, 0.60)
Q4	18.1 ± 0.4	− 0.12 (− 1.15, 0.92)	0.36 (− 0.57, 1.29)	0.35 (− 0.56, 1.26)
Q5	17.6 ± 0.4	− 0.64 (− 1.46, 0.19)	0.04 (− 0.77, 0.84)	0.04 (− 0.73, 0.81)
*P* trend	–	0.188	0.733	0.705

Model 1: adjusted for age and gender.

Model 2: adjusted for variables in Model 1 and race/ethnicity, education, household income, marital status, smoking, and alcohol consumption.

Model 3: adjusted for variables in Model 2 and sleep duration and total physical activity.

*CI* confidence interval, *NHANES* National Health and Nutrition Examination Survey, *CERAD* Consortium to Establish a Registry for Alzheimer’s Disease, *DSSS* Digit Symbol Substitution Test score, *CSP* CERAD Savings Percentage, *AFT* Animal Fluency Test.

^a^Means, standard errors, and beta estimates are weighted using sample weights.

^b^Reference group presumed as having the best temporal relationship.

We present subgroup associations between phasor magnitude and angle and cognitive scores (Model 2 alone), stratified by gender (Supplementary Table 3), age group (Supplementary Table 4), race/ethnicity (Supplementary Table 5), and education level (Supplementary Table 6). Although most *P* values for the interactions of the phasor magnitude or angle with subgroup variable were greater than 0.05, there appear to be some subgroup differences. Phasor magnitude had a stronger association with DSSS in women (b_Q1 v. Q5_ (95% CI) − 4.93 (− 7.69, − 2.17), (*P* trend, 0.007)) compared to men (b_Q1 v. Q5_ (95% CI): − 2.83 (− 6.22, 0.57), (*P* trend, 0.156)). It also had a stronger association with DSSS in those that were over 70 (b_Q1 v. Q5_ (95% CI) − 6.30 (− 9.46, − 3.13), (*P* trend, 0.002)) compared those in their 60s (b_Q1 v. Q5_ (95% CI) − 3.40 (− 6.85, 0.05), (*P* trend, 0.056)). Similarly, it had a stronger association with AFT score in the older age group (b_Q1 v. Q5_ (95% CI) − 1.40 (− 2.51, − 0.30), (*P* trend, 0.011)) compared to in the younger age group (b_Q1 v. Q5_ (95% CI) − 1.15 (− 1.12, 0.81), (*P* trend, 0.611)). Additionally, a significant association of phasor magnitude with DSSS was only observed among white participants (b_Q1 v. Q5_ (95% CI) − 4.35 (− 7.22, − 1.48), (*P* trend, 0.024)) and college graduates (b_Q1 v. Q5_ (95% CI) − 6.28 (− 10.63, − 1.92), (*P* trend, 0.003)).

## Discussion

In this nationally representative sample of older American men and women, we found that reduced strength of rest-activity and dark-light cycle coupling is associated with lower processing speed and working memory (as indicated by DSSS), which is consistent with our hypothesis. Further, the results did not show associations between coupling strength and cognitive measures of information retention and verbal fluency. Additionally, a delayed rest-activity cycle relative to dark–light cycle was not associated with any cognition outcomes when accounting for major sociodemographic factors. Finally, there was suggestive evidence for differences in the associations of phasor magnitude and DSSS among sociodemographic subgroups.

To our knowledge, only Blackwell et al.^27^ have analyzed the relationship between these phasor variables (i.e., phasor magnitude and phasor angle) and cognitive functioning. Blackwell’s analysis focused on older and predominantly white and college-educated men enrolled in the MrOS Sleep Study. They found that lower phasor magnitude was associated with reduced executive function as determined by the Trails B test^27^. Specifically, at baseline, men in the lowest quartile of phasor magnitude completed the test in 123 s, while men in the highest quartile finished in 111 s (in a model adjusting for clinical center, age, race, education level, and season). However, this association became non-significant when adjusting for further conditions and lifestyle factors. After an average of 4.2 years of follow up, the fully adjusted model showed a significant 7-s average increase in test completion time per standard deviation decrease in magnitude^27^. These findings are consistent with ours, which showed an association between a lower phasor magnitude and worse DSSS^27^. Blackwell’s findings provide further evidence that circadian entrainment affects cognitive performance. Sun et al., also using the NHANES data, investigated the relationships between rest-activity rhythm metrics and cognitive function in the elderly population. Consolidated rest-activity rhythms were significantly associated with lower cognitive scores across different domains^33^. These results are consistent with the associations found in our analysis, which established that phasor magnitude as a measure of circadian alignment is an important predictor of cognitive functioning.

Our results regarding DSSS are also supported by literature regarding the cognitive effects of circadian disruption in those subjected to jet lag and nightshift schedules^7,8,11,12^. Overall, these studies along with ours elucidate the importance of normal sleep–wake schedules that are more strongly aligned with light and dark cues from the environment for better cognition^11,12,27,33^.

While this association held true when controlling for many key demographic factors, the association became non-significant when we accounted for sleep duration and physical activity. We then examined the relationship between phasor magnitude/angle and DSSS stratified by sleep duration categories, (< 7 h, 7–9 h, and 9+ hours) and physical activity level categories (< 5000 MIMS, 5000–10,000 MIMS, and 10,000+ MIMS) and did not observe any differences in the association (Supplementary Tables 7, 8). Physical activity and sleep duration may be both drivers and consequences of misalignment between light and activity cycles, and therefore may be mediators in the association we determined in Model 2.

The present results are consistent with the literature. A meta-analysis done by Zhu et al. that examined phototherapy’s effect on cognition problems indicated that phototherapy interventions used on subjects with dementia were useful in improving attention, executive function, and working memory^34^. Using the NHANES data set, Shi and Chen investigated the relationship between ambient light (as calculated from an actigraph device) and cognitive impairment, and found that an increase in ambient light was associated with a decrease in cognitive impairment^35^. Clinically, the findings from our study and others suggest that interventions that focus on improving the alignment between dark-light exposure and rest-activity may be beneficial to cognitive health, a hypothesis that warrants further investigation in future studies.

There were no significant associations between phasor angle and cognitive scores in the analysis when we controlled for important sociodemographic and lifestyle factors. These findings are also supported by Blackwell et al. who determined that, despite a positive association between phasor magnitude and executive function, phasor angle as a predictor did not show an association with this category of cognition^27^. The lack of evidence that we and others have found that would suggest phasor angle as a risk factor for poor cognitive function may be because phasor angle reflects the timing of activity with respect to light, but not necessarily circadian entrainment.

Although we did not identify statistically significant modifying effects of age, gender, race/ethnicity, and education, there was suggestive evidence that warrants future investigations focusing on elucidating the relationship between circadian function and cognitive outcomes in different sociodemographic subgroups. Some previous studies have also investigated the relationship between circadian rhythms and cognition across different population subgroups. Santhi et al. found that long awake time combined with adverse circadian phase had a larger effect on cognitive accuracy in women compared to men^36^. Furthermore, Rabinowitz et al. corroborated our findings of stronger associations between circadian rest-activity rhythms and cognitive decline in women and older age groups; however, they found a stronger association in Black participants while we found a stronger association in white participants^37^. An interesting finding from Table 1 is worth noting. While the percentage of non-Hispanic whites remains constant between quintile 1 and quintile 5 (14% drop), the percentage of non-Hispanic Blacks drops considerably from quintile 1 to quintile 5 (74% drop). This same trend was observed when we used sleep quintiles (Supplementary Tables 9, 10), showing a strong correlation between sleep duration and phasor magnitude.

There are many strengths to our study. This paper used data from a large national survey that included both men and women as well as participants from diverse racial and socioeconomic backgrounds. This suggests that phasor magnitude may serve as a predictor of cognitive decline across many sociodemographic groups. Additionally, the type of phasor analysis calculated by our group is a novel way of quantifying alignment from actigraphy measurements and is useful in predicting cognition as well as other health outcomes. A previous study from our group that calculated phasor magnitude and angle from NHANES data found significant associations with diabetes status and glucose metabolism^38^. Further, this study may have clinical implications for which interventions can be utilized to focus on promoting circadian entrainment. Such measures can potentially improve processing speed and working memory in aging individuals.

There are also a few limitations to address regarding this analysis. First, NHANES survey data is cross-sectional rather than prospective, so we cannot make claims about the temporality of the factors being analyzed. Second, there is a lack of information on work schedules in NHANES that could provide evidence of potentially confounding effects of day vs. nightshift work. Third, regarding light measurements, the devices worn by participants are uncalibrated and placed on the wrist, which may result in over or underestimation of light exposures experienced at the cornea. Additionally, the devices do not provide any information on light spectrum, which limits our ability to accurately assess light exposure in the context of circadian response. Fourth, there is a possibility of a non-linear association between the phasor angle and cognitive test scores, which can be assessed in future studies. Lastly, there is a possibility of residual confounding present from imperfect measurements of the data that we are not able to reasonably account for, which can cause bias or affect regression estimates.

In conclusion, findings from this analysis suggest that increased rest-activity and dark-light cycle coupling strength is associated with better processing speed and working memory. If confirmed by future studies, particularly studies in prospective cohorts, this finding may motivate the development of preventative measures or therapies aimed at improving the alignment between dark-light patterns with rest-activity schedules, which may lead to cognitive benefits in older adults.

## Methods

### Sample

The NHANES is a cross-sectional survey conducted by the Centers for Disease Control and Prevention. It is designed to assess the health and nutritional status of adults and children in the US population and aims to be utilized for health promotion and disease prevention. Since 1999, the NHANES has become a continuous survey with data released every 2 years. It is comprised of interviews, physical examinations, and laboratory assays. The current analysis sample used NHANES data from 2011–2012 to 2013–2014, when the actigraph was used to measure light exposure and activity levels. The NHANES is approved by the National Center for Health Statistics Ethics Review Board^39^.

**Figure 1:**
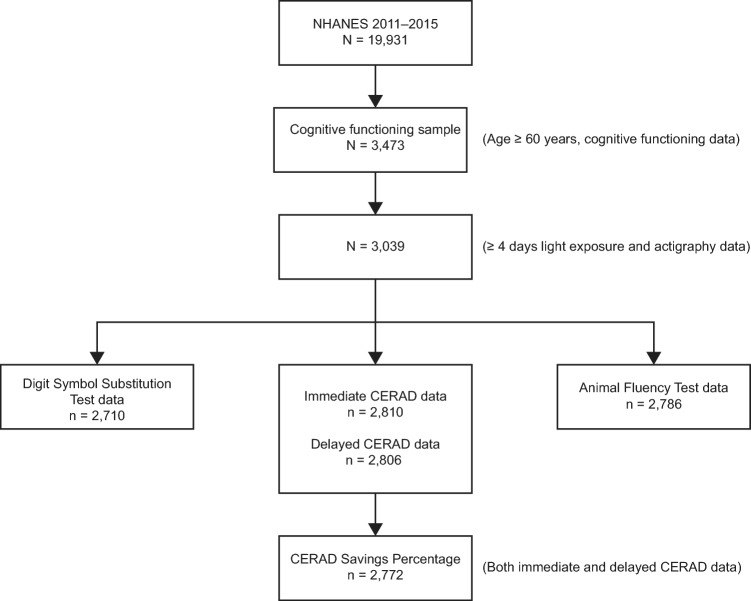
Flow chart showing the progression of participants to derive samples for Digit Symbol Substitution Test score, CERAD Savings Percentage, and Animal Fluency Test score. *NHANES* National Health and Nutrition Examination Survey, *CERAD* Consortium to Establish a Registry for Alzheimer’s Disease.

The process for sample size selection used for this paper’s analysis is presented in Fig. 1. The NHANES 2011–2014 survey included a total of 19,931 participants. The cognitive assessment module was performed among those who were 60 or older, and a total of 3473 participants provided data on cognitive functions. Of these, we excluded those that did not have light and/or activity data or had less than four valid days of actigraphy data (N = 434). Of the remaining participants, analyses for Digit Symbol Substitution Test score (DSSS), CERAD Savings Percentage (CSP), and Animal Fluency Test (AFT) score included 2710, 2772, and 2786 participants, respectively.

### Phasor analyses

Phasor analysis can be used to quantify the relationship between a person’s 24-h dark-light exposure cycle (i.e., the input to the circadian clock), and their 24-h rest-activity cycle (i.e., the behavioral output)^40,41^. Phasor magnitude represents the strength of the dark-light and rest-activity coupling exhibited by each individual, with greater values (i.e., greater phasor magnitude) representing better coupling. Phasor angle represents the temporal relationship between the 24-h dark-light and the rest-activity cycles. A more positive phasor angle indicates the rest-activity cycle was delayed relative to the dark-light cycle, and vice versa. Light exposure affects the human circadian system in a non-linear manner^42^. While spectral power distribution data were not available for the dark-light cycles the participants experienced, we applied a logistic transformation of the measured light. Further, the circadian system has a saturation level^43^. Here, we assumed a saturation level of 10,000 lx and recorded light levels exceeding this threshold were set to 10,000 lx. The dark-light and rest-activity signals were modeled with 24-h cosine functions. The phasor angle is similar, but not identical, to the difference between the rest-activity acrophase and the dark-light acrophase.

### Cognitive outcomes

The Cognitive Functioning tests in the 2011–2012 and 2013–2014 NHANES were administered by trained interviewers at the end of the NHANES face-to-face private interviews, and included the DSST, the CERAD test, and the AFT.

The DSST is conducted using a paper form with a key at the top containing 9 numbers paired with symbols^44^. Participants have 2 min to copy the corresponding symbols in 133 boxes that adjoin the numbers. The primary outcome based on this test is the DSSS, representing the total number of correct matches. This test probes processing speed, sustained attention, and working memory^28^.

The CERAD Word Learning test comprises 3 consecutive learning trials and a delayed recall trial. For each of the 3 learning trials, participants read aloud 10 words, one at a time, as the words were presented to them. Immediately after each presentation of the 10 words, participants were asked to recall as many words as possible. In each of the 3 learning trials, the order of the 10 words changed. The delayed word recall test (“Delayed Score”) was later administered after the other 2 other cognitive tests (DSST and AFT) were completed. Scores were determined as the number of correct words out of 10 for each trial. We calculated CSP as (Delayed Score/Trial 3 Score) × 100%. Although CSP was the primary outcome based on the CERAD test, we also performed a sensitivity analysis focusing on the sum of the immediate and the delayed recall scores separately. CERAD Word Learning assessments are generally used to evaluate memory for those at risk of AD^29,30^. The CSP specifically evaluates information retention^31^.

In the AFT, participants are asked to name as many animals as they can in one minute. Their AFT score is determined as one point for each animal named. This test examines categorical verbal fluency^32^. Such scores have been used to distinguish whether individuals have normal cognitive function, mild cognitive impairment, or more severe forms of impairment such as Alzheimer’s disease^45–47^.

### Covariates

Key covariates for the analysis included sociodemographic variables (gender, age, ethnicity, education level, household income, and marital status) and lifestyle factors (smoking and alcohol status, sleep duration, and total physical activity). It was hypothesized that these variables would be related to cognition. Sociodemographic variables and smoking and alcohol intake were measured by in-person interviews. Sleep duration and total physical activity were measured based on actigraphy data. Specifically, sleep duration was measured as the total minutes per day categorized as sleep and total physical activity measured as the daily sum of per-minute Monitor-Independent Movement Summary values (MIMS), both calculated as daily averages across the recording period.

### Statistical analysis

Phasor magnitude and phasor angle were divided into quintiles, with the groups presumed as having the best light–dark and activity-rest coupling (magnitude) or best temporal relationship (angle) serving as the reference. Q5 was used for phasor magnitude because it represents the larger phasor magnitude range, indicating the greatest coupling and Q1 was used for phasor angle because it represents the best temporal relationship between the light–dark and activity-rest cycles.

Descriptive statistics are presented as mean and standard error for continuous variables and percentages for categorical variables under each quintile of phasor magnitude (Table 1) and phasor angle (Table 2). These tables also contain *P* values for ANOVA tests comparing continuous variables and X^2^ tests comparing categorical variables across quintiles of phasor magnitude and angle. To determine the association of quintiles of phasor magnitude and angle with cognition scores, multiple linear regression was performed to calculate beta coefficients and 95% confidence intervals (CI).

We ran a series of models: First, the minimal model (Model 1), which we adjusted for age (continuous) and gender (men, women). In the main model (Model 2), we adjusted for multiple additional confounders including race/ethnicity (non-Hispanic white, non-Hispanic Black, Hispanic, others), education (less than high school, high school graduate, some college, college graduate or above), household income (< $20,000, $20,000–44,999, $45,000–74,999, $75,000+), marital status (married, not married), smoking (current smoker, former smoker, never smoker or less than 100 cigarettes in life), and alcohol consumption (never or less than 100 drinks in life, light (0–1 a day for men and 0–0.5 a day for women), moderate (1–2 a day for men and 0.5–1 a day for women), or heavy (more than 2 a day for men and more than 1 a day for women)). In Model 3 we adjusted for all of the variables in Model 2 as well as sleep duration (< 7 h, 7–9 h, > 9 h) and total physical activity (continuous) to evaluate to what degree the association between phasor variables and cognition is explained by individual behavioral components of the rest-activity cycle (i.e., sleep and physical activity). Probability of a Type 1 error (*P* values) for trend were determined by modeling quintiles of phasor variables as continuous (i.e., 1–5 for Q1–Q5). We also performed subgroup analyses using the main model (Model 2) stratified by age, gender, ethnicity, and education level to examine differences in the effect of circadian alignment on cognition among key demographic groups. *P* values for interaction between any two factors were calculated using a Wald test to compare a model with the interaction term to one without. For all covariates, any missingness was less than 8% and was imputed as the mode for categorical variable and median for continuous variables. Sample weights, strata, and primary sampling units provided by NHANES were used in the analysis to produce accurate estimates. Analyses were performed using Stata 14 (StataCorp LLC, College Station, TX, US).

### Supplementary Information

Supplementary Tables.

## Data Availability

The NHANES datasets analyzed during the current study are available online at the Centers for Disease Control and Prevention website: https://wwwn.cdc.gov/nchs/nhanes/Default.aspx.
