# Supplementary material for: Associations between circadian alignment and cognitive functioning in a nationally representative sample of older adults
Source: Sci Rep. 2024 Jun 12;14:13509. doi: 10.1038/s41598-024-64309-9 (PMC11169347; doi:10.1038/s41598-024-64309-9)
Supplement: Supplementary file 1 — Supplementary Tables. [file 41598_2024_64309_MOESM1_ESM.docx]

***Supplementary Tables (1–10) for***

**Associations between circadian alignment and cognitive functioning in a nationally representative sample of older adults**

Sophie Leahy MPH^1^, Qian Xiao PhD^2,3^, Chris Ho Ching Yeung PhD^2^, Mariana G Figueiro PhD^1^*

^1^ Light and Health Research Center, Department of Population Health Science and Policy, Icahn School of Medicine at Mount Sinai, New York, NY

^2^ Department of Epidemiology, Human Genetics, and Environmental Sciences, School of Public Health, The University of Texas Health Science Center at Houston, Houston, TX

^3^ Center of Spatial-Temporal Modeling for Applications in Population Sciences, School of Public Health, The University of Texas Health Science Center at Houston, Houston, TX

*Corresponding author: Mariana G Figueiro, mariana.figueiro@mountsinai.org

**Supplementary Table 1.** Associations of phasor magnitude with sum immediate CERAD scores and delayed CERAD scores in NHANES, 2011-2014

|  | **Cognitive test scores^a^** | | | |
| --- | --- | --- | --- | --- |
|  |  | **Beta estimates (95% CI)** | | |
| **Phasor magnitude quintile** | **Mean ± SE** | **Model 1** | **Model 2 (main)** | **Model 3** |
|  | **Sum immediate CERAD scores** | | | |
| Q1 | 18.6 ± 0.4 | -1.01 (-1.86, -0.15) | -0.50 (-1.28, 0.28) | 0.03 (-0.73, 0.79) |
| Q2 | 19.0 ± 0.4 | -0.52 (-1.19, 0.15) | -0.18 (-0.90, 0.54) | 0.20 (-0.56, 0.96) |
| Q3 | 19.3 ± 0.3 | -0.64 (-1.46, 0.19) | -0.35 (-1.14, 0.44) | -0.06 (-0.81, 0.70) |
| Q4 | 19.8 ± 0.3 | -0.14 (-0.69, 0.41) | 0.12 (-0.42, 0.65) | 0.26 (-0.26, 0.79) |
| Q5 | 20.2 ± 0.3 | ref^b^ | ref | ref |
| *P* trend | — | 0.018 | 0.162 | 0.973 |
|  | **Delayed CERAD scores** | | | |
| Q1 | 5.7 ± 0.2 | -0.52 (-1.03, -0.01) | -0.35 (-0.83, 0.14) | -0.07 (-0.59, 0.45) |
| Q2 | 5.9 ±0.2 | -0.32 (-0.69, 0.05) | -0.22 (-0.63, 0.18) | -0.03 (-0.46, 0.41) |
| Q3 | 6.0 ± 0.1 | -0.38 (-0.81, 0.04) | -0.29 (-0.70, 0.12) | -0.14 (-0.54, 0.26) |
| Q4 | 6.4 ± 0.2 | -0.08 (-0.37, 0.21) | -0.02 (-0.32, 0.28) | 0.06 (-0.22, 0.35) |
| Q5 | 6.5 ± 0.1 | ref | ref | ref |
| *P* trend | — | 0.031 | 0.111 | 0.698 |
| ^a^ Means, standard errors, and beta estimates are weighted using sample weights.  ^b^ Reference group presumed as having the best light-dark and activity-rest coupling.  Model 1: adjusted for age and gender.  Model 2: adjusted for variables in Model 1 and race/ethnicity, education, household income, marital status, smoking, and alcohol consumption.  Model 3: adjusted for variables in model 2 and sleep duration and total physical activity.  Abbreviations: CI, confidence interval; NHANES, National Health and Nutrition Examination Survey; CERAD, Consortium to Establish a Registry for Alzheimer’s Disease | | | | |

**Supplementary Table 2.** Associations of phasor angle with sum immediate CERAD scores and delayed CERAD scores in NHANES, 2011-2014 (weighted)

|  | **Cognitive test scores^a^** | | | |
| --- | --- | --- | --- | --- |
|  |  | **Beta Estimates (95% CI)** | | |
| **Phasor angle quintile** | **Mean ± SE** | **Model 1** | **Model 2 (main)** | **Model 3** |
|  | **Sum immediate CERAD scores** | | | |
| Q1 | 19.4 ± 0.3 | ref^b^ | ref | ref |
| Q2 | 19.8 ± 0.3 | 0.09 (-0.48, 0.65) | 0.03 (-0.53, 0.58) | -0.02 (-0.57, 0.53) |
| Q3 | 19.6 ± 0.3 | -0.34 (-0.91, 0.23) | -0.33 (-0.86, 0.21) | -0.30 (-0.82, 0.21) |
| Q4 | 19.4 ± 0.3 | -0.58 (-1.31, 0.15) | -0.23 (-0.90, 0.43) | -0.25 (-0.95, 0.44) |
| Q5 | 19.2 ± 0.3 | -0.69 (-1.44, 0.06) | -0.15 (-0.90, 0.59) | -0.14 (-0.87, 0.60) |
| *P* trend | — | 0.021 | 0.420 | 0.420 |
|  | **Delayed CERAD scores** | | | |
| Q1 | 6.2±0.1 | ref | ref | ref |
| Q2 | 6.2±0.1 | -0.15 (-0.49, 0.19) | -0.17 (-0.50, 0.15) | -0.20 (-0.53, 0.12) |
| Q3 | 6.1±0.2 | -0.36 (-0.76, 0.03) | -0.36 (-0.78, 0.06) | -0.35 (-0.75, 0.06) |
| Q4 | 6.2±0.2 | -0.26 (-0.56, 0.03) | -0.14 (-0.42, 0.13) | -0.16 (-0.44, 0.13) |
| Q5 | 6.1±0.1 | -0.31 (-0.70, 0.07) | -0.13 (-0.53, 0.26) | -0.12 (-0.51, 0.26) |
| *P* trend | — | 0.044 | 0.436 | 0.468 |
| ^a^ Means, standard errors, and beta estimates are weighted using sample weights.  ^b^ Reference group presumed as having the best temporal relationship.  Model 1: adjusted for age and gender.  Model 2: adjusted for variables in Model 1 and race/ethnicity, education, household income, marital status, smoking, and alcohol consumption.  Model 3: adjusted for variables in model 2 and sleep duration and total physical activity.  Abbreviations: CI, confidence interval; NHANES, National Health and Nutrition Examination Survey; CERAD, Consortium to Establish a Registry for Alzheimer’s Disease | | | | |

**Supplementary Table 3.** Associations (Beta Estimate (95% CI)) of phasor magnitude and phasor angle with cognitive test scores from Model 2 in NHANES 2011-2014, stratified by gender

| **Quintile** | **Cognitive test scores^a^** | | |
| --- | --- | --- | --- |
|  | **DSSS** | **CSP** | **AFT score** |
| **Phasor magnitude** | **Men** | | |
| Q1 | -2.83 (-6.22, 0.57) | -2.10 (-8.60, 4.39) | -0.66 (-2.11, 0.80) |
| Q2 | -1.56 (-4.43, 1.31) | 0.10 (-7.27, 7.46) | -0.18 (-1.41, 1.05) |
| Q3 | 0.43 (-2.69, 3.55) | -3.31 (-9.81, 3.19) | -0.24 (-1.49, 1.02) |
| Q4 | -1.40 (-4.62, 1.83) | -1.07 (-6.45, 4.31) | -0.49 (-1.94, 0.95) |
| Q5 | ref^b^ | ref | ref |
| *P* trend | 0.156 | 0.663 | 0.528 |
|  | **Women** | | |
| Q1 | -4.93 (-7.69, -2.17) | -3.42 (-8.10, 1.27) | -0.38 (-1.41, 0.66) |
| Q2 | -2.14 (-5.15, 0.87) | -2.51 (-8.51, 3.49) | -0.27 (-1.07, 0.52) |
| Q3 | -1.62 (-4.20, 0.97) | 0.25 (-4.39, 4.89) | -0.06 (-0.75, 0.62) |
| Q4 | -2.50 (-5.03, 0.03) | 0.40 (-4.18, 4.99) | 0.44 (-0.66, 1.54) |
| Q5 | ref | ref | ref |
| *P* trend | 0.007 | 0.122 | 0.143 |
| *P* interaction | 0.115 | 0.664 | 0.842 |
| **Phasor angle** | **Men** | | |
| Q1 | ref^c^ | ref | ref |
| Q2 | -0.91 (-3.36, 1.53) | -1.35 (-7.16, 4.45) | -0.76 (-1.79, 0.27) |
| Q3 | -1.64 (-4.30, 1.02) | -4.80 (-11.88, 2.28) | -0.77 (-1.95, 0.41) |
| Q4 | -2.32 (-5.09, 0.46) | -1.44 (-6.85, 3.97) | 0.57 (-0.67, 2.12) |
| Q5 | -0.45 (-2.93, 2.03) | -0.75 (-6.33, 4.83) | -0.41 (-1.66, 0.84) |
| *P* trend | 0.212 | 0.596 | 0.839 |
|  | **Women** | | |
| Q1 | ref | ref | ref |
| Q2 | -1.90 (-4.72, 0.91) | -4.34 (-8.76, 0.08) | 0.78 (-0.20, 1.75) |
| Q3 | -2.37 (-4.96, 0.22) | -2.23 (-7.84, 3.38) | 0.46 (-0.60, 1.52) |
| Q4 | -2.09 (-5.06, 0.88) | -0.50 (-4.82, 3.83) | 0.37 (-0.49, 1.23) |
| Q5 | -2.02 (-5.03, 1.00) | -1.04 (-5.09, 3.01) | 0.62 (-0.46, 1.71) |
| *P* trend | 0.233 | 0.583 | 0.508 |
| *P* interaction | 0.758 | 0.460 | 0.963 |
| ^a^ Beta estimates are weighted using sample weights and are adjusted for age, race/ethnicity, education, household income, marital status, smoking, and alcohol consumption.  ^b^ Reference group presumed as having the best light-dark and activity-rest coupling.  ^c^ Reference group presumed as having the best temporal relationship.  Abbreviations: CI, confidence interval; NHANES, National Health and Nutrition Examination Survey; CERAD, Consortium to Establish a Registry for Alzheimer’s Disease; DSSS, Digit Symbol Substitution Test score; CSP, CERAD Savings Percentage; AFT, Animal Fluency Test | | | |

**Supplementary Table 4.** Associations (Beta Estimate (95% CI)) of phasor magnitude and phasor angle with cognitive test scores from Model 2 in NHANES 2011-2014, stratified by age

| **Quintile** | **Cognitive test scores^a^** | | |
| --- | --- | --- | --- |
|  | **DSSS** | **CSP** | **AFT score** |
| **Phasor magnitude** | **Age 60-69** | | |
| Q1 | -3.40 (-6.85, 0.05) | -2.04 (-8.21, 4.12) | -0.15 (-1.12, 0.81) |
| Q2 | -3.00 (-6.44, 0.43) | -3.52 (-9.94, 2.90) | -0.04 (-1.13, 1.04) |
| Q3 | -0.52 (-3.60, 2.56) | 0.93(-4.24, 6.11) | -0.50 (-1.44, 0.43) |
| Q4 | -2.44 (-5.56, 0.68) | -0.04 (-3.28, 3.20) | 0.10 (-0.69, 0.90) |
| Q5 | ref^b^ | ref | ref |
| *P* trend | 0.056 | 0.399 | 0.611 |
|  | **Age 70-80** | | |
| Q1 | -6.30 (-9.46, -3.13) | -6.35 (-12.26, -0.45) | -1.40 (-2.51, -0.30) |
| Q2 | -1.97 (-5.49, 1.55) | -2.16 (-7.00, 2.68) | -0.82 (-1.80, 1.60) |
| Q3 | -1.73 (-4.10, 0.64) | -6.93 (-12.88, -0.98) | 0.02 (-0.82, 0.87) |
| Q4 | -1.97 (-5.41, 1.47) | -2.14 (-6.86, 2.58) | -0.23 (-1.29, 0.82) |
| Q5 | ref | ref | ref |
| *P* trend | 0.002 | 0.062 | 0.011 |
| *P* interaction | 0.181 | 0.284 | 0.172 |
| **Phasor angle** | **Age 60-69** | | |
| Q1 | ref^c^ | ref | ref |
| Q2 | -3.04 (-6.25, 0.17) | -4.82 (-9.49, -0.15) | -0.32 (-1.64, 1.00) |
| Q3 | -3.87 (-7.13, -0.60) | -4.95 (-10.20, 0.30) | -0.63 (-1.71, 0.44) |
| Q4 | -2.54 (-5.55, 0.47) | -3.68 (-7.90, 0.54) | 0.08 (-1.24, 1.39) |
| Q5 | -0.89 (-3.61, 1.83) | -4.58 (-8.71, -0.45) | -0.22 (-1.48, 1.04) |
| *P* trend | 0.666 | 0.063 | 0.981 |
|  | **Age 70-80** | | |
| Q1 | ref | ref | ref |
| Q2 | 0.21 (-2.42, 2.83) | -0.53 (-5.00, 3.95) | 0.52 (-0.32, 1.37) |
| Q3 | 0.41 (-2.28, 3.09) | -1.27 (-7.85, 5.31) | 0.44 (-0.62, 1.49) |
| Q4 | -0.91 (-3.52, 1.70) | 3.10 (-2.68, 8.88) | 0.82 (-0.32, 1.97) |
| Q5 | -1.71 (-4.06, 0.65) | 3.55 (-2.17, 9.26) | 0.50 (-0.50, 1.50) |
| *P* trend | 0.145 | 0.152 | 0.293 |
| *P* interaction | 0.482 | 0.023 | 0.398 |
| ^a^ Beta estimates are weighted using sample weights and are adjusted for gender, race/ethnicity, education, household income, marital status, smoking, and alcohol consumption.  ^b^ Reference group presumed as having the best light-dark and activity-rest coupling.  ^c^ Reference group presumed as having the best temporal relationship.  Abbreviations: CI, confidence interval; NHANES, National Health and Nutrition Examination Survey; CERAD, Consortium to Establish a Registry for Alzheimer’s Disease; DSSS, Digit Symbol Substitution Test score; CSP, CERAD Savings Percentage; AFT, Animal Fluency Test | | | |

**Supplementary Table 5.** Associations (Beta Estimate (95% CI)) of phasor magnitude and phasor angle with cognitive test scores from Model 2 in NHANES 2011-2014, stratified by race/ethnicity

| **Quintile** | **Cognitive test scores^a^** | | | |
| --- | --- | --- | --- | --- |
|  | **DSSS** | | **CSP** | **AFT score** |
| **Phasor magnitude** | **Non-Hispanic white** | | | |
| Q1 | -4.35 (-7.22, -1.48) | | -2.54 (-7.54, 2.46) | -0.60 (-1.52, 0.33) |
| Q2 | -2.15 (-5.29, 1.00) | | -2.25 (-7.27, 2.78) | -0.47 (-1.33, 0.40) |
| Q3 | -0.91 (-3.61, 1.79) | | -1.96 (-6.57, 2.65) | -0.43 (-129, 0.44) |
| Q4 | -2.44 (-5.10, 0.23) | | -0.61 (-3.95, 2.72) | 0.01 (-0.86, 0.88) |
| Q5 | ref^b^ | | ref | ref |
| *P* trend | 0.024 | | 0.276 | 0.113 |
|  | | **Non-Hispanic Black** | | |
| Q1 | 0.08 (-3.67, 3.83) | | -5.11 (-13.27, 3.06) | 0.76 (-0.61, 2.13) |
| Q2 | 0.35 (-2.76, 3.47) | | 2.18 (-4.13, 8.50) | 0.59 (-1.02, 2.20) |
| Q3 | 1.42 (-1.34, 4.17) | | 0.53 (-7.07, 8.12) | 1.47 (0.12, 2.82 |
| Q4 | 1.50 (-2.31, 5.31) | | 1.24 (-6.26, 8.74) | 0.61 (-0.80, 2.01) |
| Q5 | ref | | ref | ref |
| *P* trend | 0.534 | | 0.190 | 0.530 |
|  | | **Hispanic** | | |
| Q1 | -1.47 (-6.61, 3.67) | | 0.19 (-6.64, 7.02) | -0.07 (-1.53, 1.39) |
| Q2 | 1.66 (-1.55, 4.88) | | -0.28 (-7.41, 6.84) | 0.29 (-1.16, 1.73) |
| Q3 | 0.09 (-3.25, 3.44) | | -1.09 (-7.34, 5.16) | 0.40 (-0.47, 1.27) |
| Q4 | -0.06 (-3.11, 2.98) | | 3.72 (-2.28, 9.73) | -0.04 (-1.49, 1.41) |
| Q5 | ref | | ref | ref |
| *P* trend | 0.961 | | 0.597 | 0.776 |
|  | | **Other** | | |
| Q1 | -3.57 (-10.20, 3.05) | | -3.13 (-13.89, 7.63) | 0.61 (-1.52, 2.74) |
| Q2 | 1.12 (-3.04, 5.28) | | 2.26 (-7.45, 11.98) | 2.97 (0.71, 5.23) |
| Q3 | 3.62 (-3.32, 10.55) | | 1.23 (-8.90, 11.36) | 2.35 (-0.30, 5.01) |
| Q4 | 2.51 (-3.07, 8.09) | | -2.27 (-13.21, 8.68) | 2.88 (0.78, 4.97) |
| Q5 | ref | | ref | ref |
| *P* trend | 0.078 | | 0.966 | 0.800 |
| *P* interaction | 0.300 | | 0.820 | 0.313 |
| **Phasor angle** | **Non-Hispanic white** | | | |
| Q1 | ref^c^ | | ref | ref |
| Q2 | -1.55 (-3.64, 0.55) | | -2.89 (-7.42, 1.65) | 0.21 (-0.70, 1.12) |
| Q3 | -2.43 (-4.54, -0.32) | | -4.28 (-9.76, 1.20) | -0.04 (-0.86, 0.77) |
| Q4 | -2.41 (-4.65, -0.16) | | -1.29 (-5.85, 3.29) | 0.66 (-0.50, 1.83) |
| Q5 | -0.98 (-2.88, 0.93) | | -0.60 (-5.83, 4.64) | 0.20 (-0.96, 1.36) |
| *P* trend | 0.124 | | 0.845 | 0.529 |
|  | | **Non-Hispanic Black** | | |
| Q1 | ref | | ref | ref |
| Q2 | 0.71 (-3.18, 4.59) | | -0.68 (-8.37, 7.01) | -0.62 (-1.66, 0.42) |
| Q3 | 2.36 (-1.08, 5.79) | | 1.36 (-6.31, 9.04) | 0.34 (-0.63, 1.31) |
| Q4 | -0.88 (-4.25, 2.50) | | -0.05 (-7.98, 7.87) | 0.15 (-1.33, 1.63) |
| Q5 | -0.96 (-3.92, 2.01) | | -4.65 (-11.41, 2.10) | -0.02 (-1.23, 1.18) |
| *P* trend | 0.371 | | 0.233 | 0.660 |
|  | | **Hispanic** | | |
| Q1 | ref | | ref | ref |
| Q2 | -1.53 (-6.03, 2.97) | | 3.64 (-4.62, 11.90) | 1.08 (-0.30, 2.46) |
| Q3 | -1.42 (-5.62, 3.52) | | 1.76 (-10.08, 13.61) | 0.11 (-1.89, 2.11) |
| Q4 | -1.64 (-6.34, 4.26) | | 2.71 (-8.54, 13.96) | 0.67 (-0.87, 2.22) |
| Q5 | -3.60 (-6.58, 0.96) | | 1.65 (-6.24, 9.54) | 0.40 (-0.82, 1.61) |
| *P* trend | 0.188 | | 0.911 | 0.904 |
|  | | **Other** | | |
| Q1 | ref | | ref | ref |
| Q2 | -4.36 (-9.32, 0.60) | | -18.31 (-35.31, -1.30) | -1.66 (-5.02, 1.69) |
| Q3 | -4.71 (-10.35, 0.93) | | -7.98 (-20.02, 4.06) | -3.26 (-6.04 -0.49) |
| Q4 | -1.58 (-7.51, 4.36) | | -7.49 (-21.75, 6.77) | -2.64 (-5.38, 0.10) |
| Q5 | -2.43 (-6.85, 1.98) | | -7.48 (-23.08, 8.11) | -1.79 (-4.11, 0.52) |
| *P* trend | 0.746 | | 0.704 | 0.127 |
| *P* interaction | 0.863 | | 0.778 | 0.192 |
| ^a^ Beta estimates are weighted using sample weights and are adjusted for gender, age, education, household income, marital status, smoking, and alcohol consumption.  ^b^ Reference group presumed as having the best light-dark and activity-rest coupling.  ^c^ Reference group presumed as having the best temporal relationship.  Abbreviations: CI, confidence interval; NHANES, National Health and Nutrition Examination Survey; CERAD, Consortium to Establish a Registry for Alzheimer’s Disease; DSSS, Digit Symbol Substitution Test score; CSP, CERAD Savings Percentage; AFT, Animal Fluency Test | | | | |

**Supplementary Table 6.** Associations (beta estimate (95% CI)) of phasor magnitude and phasor angle with cognitive test scores from Model 2 in NHANES 2011-2014, stratified by education

| **Quintile** | **Cognitive test scores^a^** | | |
| --- | --- | --- | --- |
|  | **DSSS** | **CSP** | **AFT score** |
| **Phasor magnitude** | **Less than high school** | | |
| Q1 | -0.80 (-4.96, 3.36) | -2.00 (-9.93, 5.92) | -0.26 (-1.51, 0.99) |
| Q2 | 0.12 (-4.08, 4.33) | 0.58 (-6.20, 7.36) | -0.04 (-1.48, 1.39) |
| Q3 | 0.21 (-3.34, 3.76) | -0.85 (-7.41, 5.70) | 0.93 (-0.60, 2.47) |
| Q4 | 0.05 (-3.06, 3.16) | -3.05 (-9.59, 3.50) | 0.65 (-0.57, 1.86) |
| Q5 | ref^b^ | ref | ref |
| *P* trend | 0.743 | 0.970 | 0.436 |
|  | **High school graduate** | | |
| Q1 | -4.39 (-11.00, 2.21) | -4.89 (-12.04, 2.25) | -0.08 (-1.13, 0.96) |
| Q2 | -1.38 (-5.81, 3.06) | -8.99 (-15.42, -2.55) | -0.29 (-1.26, 0.68) |
| Q3 | -1.54 (-5.83, 2.75) | -6.08 (-13.62, 1.47) | 0.77 (-0.26, 1.79) |
| Q4 | -2.18 (-8.57, 4.20) | -3.94 (-8.50, 0.62) | 1.64 (0..19, 3.10) |
| Q5 | ref | ref | ref |
| *P* trend | 0.209 | 0.069 | 0.238 |
|  | **Some college** | | |
| Q1 | -3.29 (-6.40, -0.18) | -3.00 (-12.59, 6.60) | -0.05 (-1.68, 1.59) |
| Q2 | -1.84 (-5.56, 1.87) | -2.01 (-10.47, 6.46) | -0.37 (-1.87, 1.13) |
| Q3 | -1.50 (-4.94, 1.94) | 2.34 (-5.68, 10.37) | -0.81 (-1.97, 0.35) |
| Q4 | -5.43(-8.10, -2.75) | 0.78 (-6.71, 8.27) | -0.24 (-1.35, 0.86) |
| Q5 | ref | ref | ref |
| *P* trend | 0.382 | 0.505 | 0.776 |
|  | **College graduate or above** | | |
| Q1 | -6.28 (-10.63, -1.92) | -1.39 (-9.62, 6.83) | -1.07 (-2.65, 0.51) |
| Q2 | -3.69 (-7.67, 0.29) | 3.88 (-4.12, 11.88) | -0.34 (-2.32, 1.63) |
| Q3 | 0.06 (-3.78, 3.89) | -1.55 (-9.27, 6.17) | -0.35 (-2.15, 1.46) |
| Q4 | 0.84 (-2.68, 4.36) | 2.84 (-3.09, 8.77) | -0.77 (-2.77, 1.22) |
| Q5 | ref | ref | ref |
| *P* trend | 0.003 | 0.941 | 0.431 |
| *P* interaction | 0.197 | 0.778 | 0.948 |
| **Phasor angle** | **Less than high school** | | |
| Q1 | ref^c^ | ref | ref |
| Q2 | 4.25 (0.19, 8.31) | -0.004 (-6.15, 6.14) | 1.24 (0.01, 2.48) |
| Q3 | 2.56 (-1.25, 6.38) | -0.25 (-8.52, 8.02) | 1.61 (0.15, 3.06) |
| Q4 | 0.73 (-2.63, 4.10) | 4.80 (-2.55, 12.15) | 1.42 (0.42, 2.42) |
| Q5 | 0.66 (-2.76, 4.08) | 2.30 (-3.84, 8.45) | 1.05 (-0.10, 2.20) |
| *P* trend | 0.348 | 0.146 | 0.099 |
|  | **High school graduate** | | |
| Q1 | ref | ref | ref |
| Q2 | -0.68 (-4.43, 3.06) | -0.66 (5.64, 4.32) | -0.53 (-1.43, 0.36) |
| Q3 | -0.01 (-4.81, 4.79) | -2.70 (-9.29, 3.88) | 0.17 (-0.80, 1.14) |
| Q4 | -1.61 (-6.70, 3.49) | -2.35 (-8.28, 3.59) | -0.23 (-1.95, 1.49) |
| Q5 | 2.35 (-1.10, 5.80) | -2.06 (-7.24, 3.13) | 1.14 (-0.39, 2.68) |
| *P* trend | 0.575 | 0.296 | 0.264 |
|  | **Some college** | | |
| Q1 | ref | ref | ref |
| Q2 | -3.18 (-7.43, 1.08) | -3.96 (-12.26, 4.33) | 0.11 (-1.44, 1.66) |
| Q3 | -3.47 (-7.44, 0.50) | -0.98 (-10.60, 8.64) | -0.42 (-2.12, 1.27) |
| Q4 | -1.62 (-5.97, 2.73) | 1.75 (-5.72, 9.21) | 0.78 (-1.09, 2.66) |
| Q5 | -1.88 (-6.28, 2.53) | -1.15 (-10.48, 8.17) | -0.46 (-2.01, 1.09) |
| *P* trend | 0.677 | 0.614 | 0.942 |
|  | **College graduate or above** | | |
| Q1 | ref | ref | ref |
| Q2 | -2.04 (-5.45, 1.37) | -4.77 (-9.03, -0.51) | -0.03 (-1.60, 1.54) |
| Q3 | -3.67 (-7.28, -0.06) | -7.98 (-15.20, -0.77) | -0.57 (-2.29, 1.16) |
| Q4 | -3.69 (-8.01, 0.64) | -5.57 (-12.21, 1.07) | 0.36 (-1.28, 2.00) |
| Q5 | -4.31 (-7.87, -0.74) | -2.37 (-8.58, 3.85) | -0.22 (-2.17, 1.74) |
| *P* trend | 0.024 | 0.234 | 0.940 |
| *P* interaction | 0.349 | 0.373 | 0.791 |
| ^a^ Beta estimates are weighted using sample weights and are adjusted for gender, age, race/ethnicity, household income, marital status, smoking, and alcohol consumption.  ^b^ Reference group presumed as having the best light-dark and activity-rest coupling.  ^c^ Reference group presumed as having the best temporal relationship.  Abbreviations: CI, confidence interval; NHANES, National Health and Nutrition Examination Survey; CERAD, Consortium to Establish a Registry for Alzheimer’s Disease; DSSS, Digit Symbol Substitution Test score; CSP, CERAD Savings Percentage; AFT, Animal Fluency Test | | | |

**Supplementary Table 7.** Associations (beta estimate (95% CI)) of phasor magnitude and phasor angle with cognitive test scores from Model 2 in NHANES 2011-2014, stratified by sleep duration categories

| **Quintile** | **Cognitive test scores^a^** | | |
| --- | --- | --- | --- |
|  | **DSSS** | **CSP** | **AFT score** |
| **Phasor magnitude** | **<7 Hours** | | |
| Q1 | -3.34 (-5.81, -0.88) | 1.41 (-5.46, 8.27) | -0.58 (-1.70, 0.54) |
| Q2 | 0.50 (-3.03, 4.04) | 1.13 (-5.42, 7.69) | 1.31 (0.01, 2.61) |
| Q3 | -0.16 (-3.13, 2.81) | 0.84 (-4.43, 6.11) | -0.42 (-1.37, 0.54) |
| Q4 | -1.59 (-4.00, 0.62) | 0.49 (-2.27, 3.24) | -0.13 (-1.01, 0.75) |
| Q5 | ref^b^ | ref | ref |
| *P* trend | 0.193 | 0.670 | 0.796 |
|  | **7-9 Hours** | | |
| Q1 | -1.01 (-3.99, 1.97) | -2.38 (-7.12, 2.36) | 0.70 (-1.00, 2.39) |
| Q2 | -1.80 (-5.07, 1.46) | -5.17 (-10.18, -0.14) | -0.90 (-2.30, 0.51) |
| Q3 | -1.04 (-4.97, 2.88) | -4.43 (-10.94, 2.08) | -0.14 (-1.65, 1.36) |
| Q4 | -1.86 (-5.44, 1.69) | -1.40 (-6.48, 3.68) | 0.32 9 (-0.93, 1.57) |
| Q5 | ref | ref | ref |
| *P* trend | 0.397 | 0.045 | 0.875 |
|  | **9+ Hours** | | |
| Q1 | -2.49 (-8.73, 3.75) | -3.87 (-19.49, 11.76) | 1.19 (-0.74, 3.12) |
| Q2 | 2.62 (-2.93, 8.18) | 7.50 (-4.66, 19.66) | 2.43 (0.22, 4.64) |
| Q3 | 4.16 (-1.01, 9.35) | 4.56 (-9.16, 18.29) | 3.50 (1.49, 5.51) |
| Q4 | -1.36 (-9.22, 6.50) | 4.02 ( -8.95, 16.98) | 1.83 (-1.76, 5.41) |
| Q5 | ref | ref | ref |
| *P* trend | 0.584 | 0.541 | 0.727 |
| *P* interaction | 0.910 | 0.313 | 0.908 |
| **Phasor angle** | **<7 Hours** | | |
| Q1 | ref^c^ | ref | ref |
| Q2 | -2.38 (-5.68, 0.93) | -4.91 (-9.57, -0.26) | -0.03 (-1.30, 1.25) |
| Q3 | -2.70 (-6.52, 1.11) | -5.22 (-12.22, 1.78) | -0.30 (-1.33, 0.74) |
| Q4 | -1.98 (-5.25, 1.29) | -1.88 (-7.34, 3.58) | 0.33 (-1.07, 1.74) |
| Q5 | -3.37 (-6.05, -0.69) | -4.58 (-10.75, 1.59) | -0.17 (-1.66, 1.33)) |
| *P* trend | 0.061 | 0.349 | 0.976 |
|  | **7-9 Hours** | | |
| Q1 | ref | ref | ref |
| Q2 | -1.10 (-3.92, 1.72) | -4.97 (-11.18, 1.25) | 0.03 (-1.17, 1.22) |
| Q3 | -1.30 (-4.02, 1.42) | -5.65 (-10.44, -0.87) | 0.37 (-0.77, 1.51) |
| Q4 | -2.05 (-5.28, 1.18) | -2.75 (-8.94, 3.44) | 0.49 (-0.65, 1.64) |
| Q5 | -0.59 (-3.63, 2.47) | -1.04 (-6.42, 4.33) | 0.51 (-0.64, 1.66) |
| *P* trend | 0.506 | 0.831 | 0.229 |
|  | **9+ Hours** | | |
| Q1 | ref | ref | ref |
| Q2 | -2.84 (-8.83, 3.15) | 8.49 (-3.44, 20.42) | -0.53 (-3.03, 1.98) |
| Q3 | -4.90 (-10,34, 0.55) | 7.96 (-3.32, 19.24) | -2.21 (-4.51, 0.09) |
| Q4 | -8.73 (-13.29, -4.17) | 4.20 (-6.55, 14.95) | -1.94 (-4.38, 0.50) |
| Q5 | 1.07 (-5.89, 8.03) | 7.45 (-4.13, 19.02) | -1.54 (-4.24, 1.17) |
| *P* trend | 0.120 | 0.357 | 0.095 |
| *P* interaction | 0.665 | 0.384 | 0.333 |
| ^a^ Beta estimates are weighted using sample weights and are adjusted for gender, age, race/ethnicity, household income, marital status, smoking, and alcohol consumption.  ^b^ Reference group presumed as having the best light-dark and activity-rest coupling.  ^c^ Reference group presumed as having the best temporal relationship.  Abbreviations: CI, confidence interval; NHANES, National Health and Nutrition Examination Survey; CERAD, Consortium to Establish a Registry for Alzheimer’s Disease; DSSS, Digit Symbol Substitution Test score; CSP, CERAD Savings Percentage; AFT, Animal Fluency Test | | | |

**Supplementary Table 8.** Associations (beta estimate (95% CI)) of phasor magnitude and phasor angle with cognitive test scores from Model 2 in NHANES 2011-2014, stratified by physical activity categories

| **Quintile** | **Cognitive test scores^a^** | | |
| --- | --- | --- | --- |
|  | **DSSS** | **CSP** | **AFT score** |
| **Phasor magnitude** | **<5,000 MIMs** | | |
| Q1 | 5.32 (-2.48, 13.12) | -12.01 (-27.54, 3.52) | 5.43 (1.32, 9.55) |
| Q2 | 6.77 (-0.32, 13.86) | -5.31 (-20.35, 9.74) | 6.96 (1.75, 12.17) |
| Q3 | 12.03 (4.88, 19.17) | 1.44 (-11.76, 14.64) | 7.79 (3.36, 12.23) |
| Q4 | 4.11 (-3.49, 11.70) | -12.00 (-32.19, 8.18) | 7.01 (2.05, 11.97) |
| Q5 | ref^b^ | Ref | ref |
| *P* trend | 0.338 | 0.100 | 0.082 |
|  | **5,000-10,000 MIMs** | | |
| Q1 | -2.45 (-5.12, 0.21) | -3.11 (-6.88, 6.26) | -0.47 (-1.50, 0.33) |
| Q2 | -0.99 (-4.73, 2.75) | 0.09 (-6.16, 6.35) | -0.65 (-1.66, 0.36) |
| Q3 | -2.16 (-5.94, 1.63) | -3.03 (-10.18, 4.11) | -0.80 (-1.73, 0.12) |
| Q4 | -3.12 (-7.36, 1.11) | 0.06 (-5.25, 5.36) | -0.62 (-1.87, 0.64) |
| Q5 | ref | ref | ref |
| *P* trend | 0.375 | 0.932 | 0.481 |
|  | **10,000+ MIMs** | | |
| Q1 | -0.86 (-5.13, 3.40) | 2.11 (-3.84, 8.07) | 1.39 (-0.66, 3.44) |
| Q2 | 2.11 (-1.53, 5.75) | -1.40 (-7.02, 4.22) | 1.62 (-0.03, 3.27) |
| Q3 | 2.10 (-1.21, 5.41) | 1.12 (-3.17, 5.40) | 0.81 (-0.15, 1.78) |
| Q4 | -1.11 (-3.35, 1.13) | -0.47 ( -4.15, 3.22) | 0.80 (-0.30, 1.90) |
| Q5 | ref | ref | ref |
| *P* trend | 0.327 | 0.781 | 0.011 |
| *P* interaction | 0.040 | 0.050 | 0.004 |
| **Phasor angle** | **<5,000 MIMs** | | |
| Q1 | ref^c^ | ref | ref |
| Q2 | -8.47 (-14.06, -2.88) | -2.55 (-20.93, 15.83) | -0.40 (-2.55, 1.74) |
| Q3 | -5.66 (-12.32, 1.01) | 2.90 (-12.39, 18.19) | 0.55 (-2.35, 3.46) |
| Q4 | -7.65 (-18.14, 2.84) | 3.13 (-11.63, 17.90) | 0.53 (-1.85, 2.92) |
| Q5 | -3,49 (-10.36, 3.38) | -10.88 (-24.22, 2.45) | 0.63 (-1.46, 2.73) |
| *P* trend | 0.301 | 0.317 | 0.461 |
|  | **5,000-10,000 MIMs** | | |
| Q1 | ref | ref | ref |
| Q2 | -2.65 (-5.01, -0.29) | -2.85 (-7.82, 2.12) | -0.64 (-1.77, 0.48) |
| Q3 | -2.26 (-4.55, 0.04) | -3.39 (-8.25, 1.47) | -0.27 (-1.19, 0.65) |
| Q4 | -3.22 (-5.90, -0.54) | -2.85 (-7.60, 1.89) | 0.03 (-1.22, 1.27) |
| Q5 | -1.96 (-5.30, 1.38) | -0.14 (-4.11, 3.82) | -0.22 (-1.32, 0.87) |
| *P* trend | 0.229 | 0.796 | 0.889 |
|  | **10,000+ MIMs** | | |
| Q1 | ref | ref | Ref |
| Q2 | -1.68 (-4.90, 1.53) | -3.68 (-9.31, 1.96) | 0.20 (-1.37, 1.77) |
| Q3 | -2.84 (-5.61, -0.06) | -5.21 (-12.73, 2.32) | -0.76 (-1.83, 0.32) |
| Q4 | -3.12 (-6.65, 0.40) | -0.03 (-5.88, 5.82) | -0.05 (-1.29, 1.18) |
| Q5 | -1.73 (-5.88, 2.43) | -1.49 (-8.42, 5.44) | 0.10 (-1.38, 1.57) |
| *P* trend | 0.212 | 0.920 | 0.819 |
| *P* interaction | 0.895 | 0.811 | 0.478 |
| ^a^ Beta estimates are weighted using sample weights and are adjusted for gender, age, race/ethnicity, household income, marital status, smoking, and alcohol consumption.  ^b^ Reference group presumed as having the best light-dark and activity-rest coupling.  ^c^ Reference group presumed as having the best temporal relationship.  Abbreviations: CI, confidence interval; NHANES, National Health and Nutrition Examination Survey; CERAD, Consortium to Establish a Registry for Alzheimer’s Disease; DSSS, Digit Symbol Substitution Test score; CSP, CERAD Savings Percentage; AFT, Animal Fluency Test; MIMS, sum of per-minute Monitor-Independent Movement Summary values | | | |

**Supplementary Table 9.** Associations of sleep duration categories with cognitive test scores in NHANES 2011-2014

| **Sleep duration** | **Cognitive test scores^a^** | | | | |
| --- | --- | --- | --- | --- | --- |
|  | **Mean ± SE** | **Beta estimates (95% CI)** | | | |
|  |  | **Model 1** | **Model 2 (main)** | **Model 3** | **Model 4** |
|  | **DSSS** | | | | |
| <6 hours | 53.6 ± 1.0 | 3.13 (0.44, 5.81) | 3.63 (1.23, 6.04) | 2.10 (-0.59, 4.79) | 1.55 (-1.20, 4.31) |
| 6-7 hours | 54.4 ± 0.9 | 5.06 (2.59, 7.52) | 4.42 (2.31, 6.52) | 2.84 (0.65, 5.04) | 2.53 (0.26, 4.81) |
| 7-8 hours | 52.0 ± 0.8 | 4.33 (1.56, 7.10) | 3.84 (1.31, 6.37) | 2.51 (-0.08, 5.11) | 2.23 (-0.38, 4.83) |
| 8-9 hours | 51.9 ± 1.0 | 5.00 (2.19, 7.81) | 3.43 (1.02, 5.85) | 2.80 (0.22, 5.39) | 2.63 (0.05, 5.22) |
| >9 hours | 44.6 ± 1.3 | ref^b^ | ref | ref | ref |
| *P* trend | — | 0.142 | 0.012 | 0.300 | 0.536 |
|  | **CSP** | | | | |
| <6 hours | 79.4 ± 1.1 | 1.82 (-2.11, 5.76) | 2.03 (-1.83, 5.88) | 0.87 (-3.71, 5.46) | 0.31 (-4.0, 4.63) |
| 6-7 hours | 80.8 ± 1.1 | 4.09 (0.62, 7.57) | 4.17 (0.73, 7.60) | 2.97 (-1.15, 7.09) | 2.61 (-1.33, 6.56) |
| 7-8 hours | 79.8 ± 0.7 | 4.61 (0.96, 8.25) | 4.80 (1.02, 8.58) | 3.80 (-0.42, 8.02) | 3.49 (-0.47, 7.45) |
| 8-9 hours | 75.8 ± 1.3 | 1.33 (-3.07, 5.73) | 1.13 (-3.33, 5.59) | 0.64 (-3.93, 5.23) | 0.46 (-4.09, 5.00) |
| >9 hours | 72.5 ± 1.6 | ref | ref | ref | ref |
| *P* trend | — | 0.202 | 0.138 | 0.607 | 0.767 |
|  | **AFT score** | | | | |
| <6 hours | 18.6 ± 0.3 | 1.10 (-0.08, 2.28) | 1.30 (0.15, 2.45) | 0.91 (-0.29, 2.11) | 0.81 (-0.47, 2.09) |
| 6-7 hours | 18.8 ± 0.3 | 1.60 (0.62, 2.57) | 1.39 (0.43, 2.34) | 0.98 (-0.02, 1.98) | 0.93 (-0.11, 1.98) |
| 7-8 hours | 17.8 ± 0.3 | 0.94 (-0.31, 2.20) | 0.77 (-0.42, 1.96) | 0.43 (-0.85, 1.71) | 0.39 (-0.91, 1.68) |
| 8-9 hours | 17.7 ± 0.4 | 0.95 (-0.17, 2.08) | 0.64 (-0.43, 1.70) | 0.47 (-0.60, 1.54) | 0.44 (-0.65, 1.53) |
| >9 hours | 16.1 ± 0.5 | ref | ref | ref | ref |
| *P* trend | — | 0.025 | 0.008 | 0.068 | 0.119 |
| ^a^ Means, standard errors, and beta estimates are weighted using sample weights.  ^b^ Reference group presumed as having the best light-dark and activity-rest coupling.  Model 1: adjusted for age and gender.  Model 2: adjusted for variables in Model 1 and race/ethnicity, education, household income, marital status, smoking, and alcohol consumption.  Model 3: adjusted for variables in model 2 and total physical activity.  Model 4: adjusted for variables in model 2 with total physical activity and phasor magnitude.  Abbreviations: CI, confidence interval; NHANES, National Health and Nutrition Examination Survey; CERAD, Consortium to Establish a Registry for Alzheimer’s Disease; DSSS, Digit Symbol Substitution Test score; CSP, CERAD Savings Percentage; AFT, Animal Fluency Test | | | | | |

**Supplementary Table 10.** Associations of sleep duration continuous with cognitive test scores in NHANES 2011-2014

| **Sleep duration** | **Cognitive test score^a^** **beta estimates (95% CI)** | | | |
| --- | --- | --- | --- | --- |
|  | **Model 1** | **Model 2 (main)** | **Model 3** | **Model 4** |
|  | **DSSS** | | | |
| Beta estimate | -0.50 (-0.95, -0.05) | -0.60 (-0.99, -0.22) | -0.42 (-0.80, -0.05) | -0.31 (-0.73, 0.12) |
| *P* value | 0.032 | 0.003 | 0.028 | 0.151 |
|  | **CSP** | | | |
| Beta estimate | -0.41 (-1.05, 0.23) | -0.43 (-1.06, 0.20) | -0.27 (-0.95, 0.42) | -0.15 (-0.89, 0.60) |
| *P* value | *0.200* | *0.170* | *0.433* | *0.696* |
|  | **AFT score** | | | |
| Beta estimate | -0.19 (-0.35, -0.04) | -0.22 (-0.37, -0.07) | -0.17 (-0.32, -0.02) | -0.15 (-0.33, 0.03) |
| *P* value | 0.018 | 0.006 | 0.025 | 0.097 |
| ^a^ Means, standard errors, and beta estimates are weighted using sample weights.  Model 1: adjusted for age and gender.  Model 2: adjusted for variables in Model 1 and race/ethnicity, education, household income, marital status, smoking, and alcohol consumption.  Model 3: adjusted for variables in model 2 and total physical activity.  Model 4: adjusted for variables in model 2 with total physical activity and phasor magnitude.  Abbreviations: CI, confidence interval; NHANES, National Health and Nutrition Examination Survey; CERAD, Consortium to Establish a Registry for Alzheimer’s Disease; DSSS, Digit Symbol Substitution Test score; CSP, CERAD Savings Percentage; AFS, Animal Fluency Test. | | | | |
